# Machine-Learning-Assisted Design of Powder-Metallurgy Ti-Al-Nb Alloys Through Rare-Earth Microalloying: Microstructure and Mechanical Properties

**DOI:** 10.3390/ma19214568

**Published:** 2026-10-29

**Authors:** Xinze Li, Qilin Yu, Guoqing Xia, Xifeng Yan, Zhu Li, Chaoqun Luo, Qi Wang, Yaran Zhang, Qi Cai

**Affiliations:** 1 School of Materials Science and Engineering, Beijing Institute of Technology, Beijing 100081, China; 3120241037@bit.edu.cn (X.L.);; 2 Tianjin Key Laboratory of Integrated Design and On-Line Monitoring for Light Industry & Food Machinery and Equipment, School of Mechanical Engineering, Tianjin University of Science and Technology, Tianjin 300457, China; 3 Material Science Research Center, Xingtai University, Xingtai 054001, China; 202420008@xttc.edu.cn

**Keywords:** Ti-Al-Nb alloy, powder metallurgy, machine learning, rare-earth microalloying, oxygen redistribution, mechanical properties

## Abstract

Powder-metallurgy (PM) Ti-Al-Nb alloys are attractive for high-temperature structural applications but remain limited by poor room-temperature ductility. This study combines an exploratory machine-learning (ML) analysis with rare-earth (RE; La and Ce) microalloying to examine relationships among composition, thermal response, microstructure, and tensile properties. The ML dataset comprised 30 in-house Ti-Al-Nb experimental records. Four differential scanning calorimetry (DSC) descriptors—the peak areas and peak temperatures in the 800–900 °C and 1000–1100 °C ranges—were used to predict room-temperature yield strength. Six regression algorithms were compared using a 70/30 train–test split and five-fold cross-validation during hyperparameter selection. The gradient boosting model achieved the best test performance (R^2^ = 0.8752; MSE = 0.0089 on the normalized response). The model was used as a screening and interpretation aid rather than as a direct optimizer of the final sintering condition; the 1400 °C/3 h sintering schedule was an established process from prior laboratory work. For Ti-22Al-(25 − x)Nb-xRE alloys (x = 0–2 at.%), RE addition produced RE_2_O_3_-containing microstructures, reduced the mean grain size from 51.6 to 18.1 μm, and narrowed the O-phase laths from about 0.55 to 0.19 μm. At room temperature, the highest peak true stress was 572 MPa at 1.0 at.% RE, whereas the largest true fracture strain was 0.63% at 1.5 at.% RE. At 600 °C, the highest strength was 634 MPa at 0.5 at.% RE, while the largest true fracture strain was 0.98% at 1.5 at.% RE. Accordingly, 1.5 at.% RE provides the best overall strength–ductility balance across both test temperatures, although it does not maximize every individual property. The observed trends are consistent with a proposed RE–O redistribution and particle-pinning mechanism, but direct quantitative oxygen analysis and high-resolution phase mapping were not available; mechanistic statements are therefore treated as evidence-supported interpretations rather than definitive proof.

## 1. Introduction

Ti_2_AlNb-based intermetallic alloys are promising lightweight structural materials for aerospace and other elevated-temperature applications because they combine relatively low density with high specific strength, oxidation resistance, and useful creep resistance [1,2,3]. Among them, Ti-22Al-25Nb has been widely studied as a representative O-phase alloy. Its practical use, however, is constrained by limited room-temperature tensile ductility and by the sensitivity of its final properties to phase constitution, grain size, porosity, and processing history [4,5,6,7]. Composition-dependent microstructures and properties have also been investigated in γ-TiAl [8], Ta-modified Ti–6Al–7Nb and Ti–Al–Nb [9,10], and Al–Mo–Nb–Ti–V–Zr refractory alloys [11]. Cost and the balance of mechanical properties have likewise guided Ti–Al–Fe alloy design [12] and high-strength titanium development more broadly [13]. Powder metallurgy offers near-net-shape processing and avoids some of the segregation and material-loss problems associated with conventional melting and extensive forging, but PM Ti-Al-Nb alloys also experience complex reaction diffusion, intermediate-phase formation, and oxygen uptake during powder handling and sintering [6,7,14,15]. Related processing routes include precision casting [16], elemental-powder fusion [17], induction sintering [18], and powder-compact extrusion [19,20]; PM strategies emphasize control of densification and microstructure [21]. These coupled effects make composition and process optimization difficult by trial-and-error alone.

Rare-earth additions provide one possible route for microstructural control. RE additions can also alter oxidation behavior in β-stabilized TiAl, with a strong dependence on addition level [22]. La-, Ce-, and related RE-bearing additions have been reported to refine grains, form stable oxide particles, modify phase fractions, and in some Ti alloys change the distribution of interstitial oxygen. These effects can be beneficial because oxygen strongly influences phase stability and room-temperature brittleness in titanium alloys. Yan et al. reported that Y_2_O_3_ formation in YH_2_-added PM Ti-Nb-Ta alloys reduced solid-solution oxygen in the metallic phases and altered oxygen partitioning [23]. This result provides a mechanistic reference for the present La-Ce-containing alloys but does not demonstrate that the same change occurred here. In the present study, conventional SEM-EDS results from grain-boundary α_2_ and B2-matrix regions are reported as apparent oxygen contents. These values are used only for comparison within the same dataset because SEM-EDS is not sufficiently reliable for accurate light-element quantification and cannot determine bulk or exact phase-resolved oxygen concentrations. Accordingly, the RE-O effect is discussed as a plausible redistribution mechanism rather than directly proven deoxygenation.

Data-driven alloy design can complement such experiments by identifying composition-processing-property correlations that are difficult to isolate empirically. Recent reviews have shown rapid growth of ML-assisted alloy design, while also emphasizing data quality, validation strategy, interpretability, and the special challenges of small datasets [24,25]. Titanium-alloy studies have begun to use ML for high-strength alloy design, high-throughput screening, and property prediction [26,27]. These reports also indicate that model performance should be interpreted cautiously when the available experimental data are limited. Against this background, the present work uses DSC descriptors and room-temperature yield strength from an in-house Ti-Al-Nb dataset to compare six regression algorithms, then experimentally evaluates a Ti-22Al-(25 − x)Nb-xRE series. The objectives are to (i) establish a transparent ML workflow and clarify its limited role in the study, (ii) quantify the evolution of phase fractions and microstructure with RE substitution, (iii) assess room-temperature and 600 °C tensile behavior, and (iv) propose, within the limits of the available evidence, how RE-containing oxide particles and oxygen redistribution may contribute to the observed strength–ductility changes. Because RE was substituted for Nb, the simultaneous reduction in Nb content is explicitly considered when interpreting the results.

## 2. Materials and Methods

### 2.1. Data Collection and Machine Learning Guidance

The ML analysis used 30 experimental records generated within the authors’ research group rather than data mined from external databases. Each record contained four DSC descriptors and one mechanical-property target. The input variables were A_1_ (integrated peak area in the 800–900 °C range, J g^−1^), T_1_ (peak temperature in that range, °C), A_2_ (integrated peak area in the 1000–1100 °C range, J g^−1^), and T_2_ (the corresponding peak temperature, °C); the target variable was room-temperature yield strength (MPa). The dataset was randomly shuffled and divided into 70% training data (21 records) and 30% test data (9 records). Hyperparameters were selected on the training data by grid search with five-fold cross-validation using the mean validation MSE as the selection criterion. The workflow used random_state = 42 for the subsequent data split, cross-validation, and stochastic estimators where applicable; however, the initial shuffle was not assigned a fixed seed. The absence of a fixed seed limits exact reproduction of the original test set, so the ML results are treated as exploratory rather than universally generalizable.

Six regression algorithms were evaluated: support vector regression (SVR), elastic net (EN), a feed-forward deep neural network (DNN), k-nearest neighbors regression (KNN), random forest (RF), and gradient boosting (GB). SVR provides a margin-based linear/nonlinear regression framework; EN is a regularized linear model; KNN predicts from local neighboring samples; RF averages decorrelated decision trees; GB sequentially fits trees to residual errors; and the DNN represents nonlinear feature interactions through hidden layers. Inputs were scaled using Min–Max normalization followed by Z-score standardization, and the response was Min–Max-normalized for model fitting. The reported MSE therefore applies to the normalized response and is not expressed in MPa^2^, whereas R^2^ remains dimensionless. Because preprocessing choices can affect comparisons of scale-sensitive models in small datasets, the relative ranking is used primarily to identify a practical screening model. Table 1 lists the hyperparameter search ranges and selected configurations. The selected GB and RF configurations both use 200 trees with a maximum depth of 3, combining multiple learners while limiting the complexity of individual trees. For GB, the learning rate of 0.1 scales the contribution of each boosting stage. The DNN uses two hidden layers with four neurons each and tanh activation. SVR uses a linear kernel, while EN combines L1 and L2 regularization with an L1 mixing ratio of 0.9. KNN uses four equally weighted neighbors and Manhattan distance (*p* = 1). These settings were selected by minimizing the mean validation MSE within the specified search spaces and were then used for test-set evaluation.

**Figure 1:**
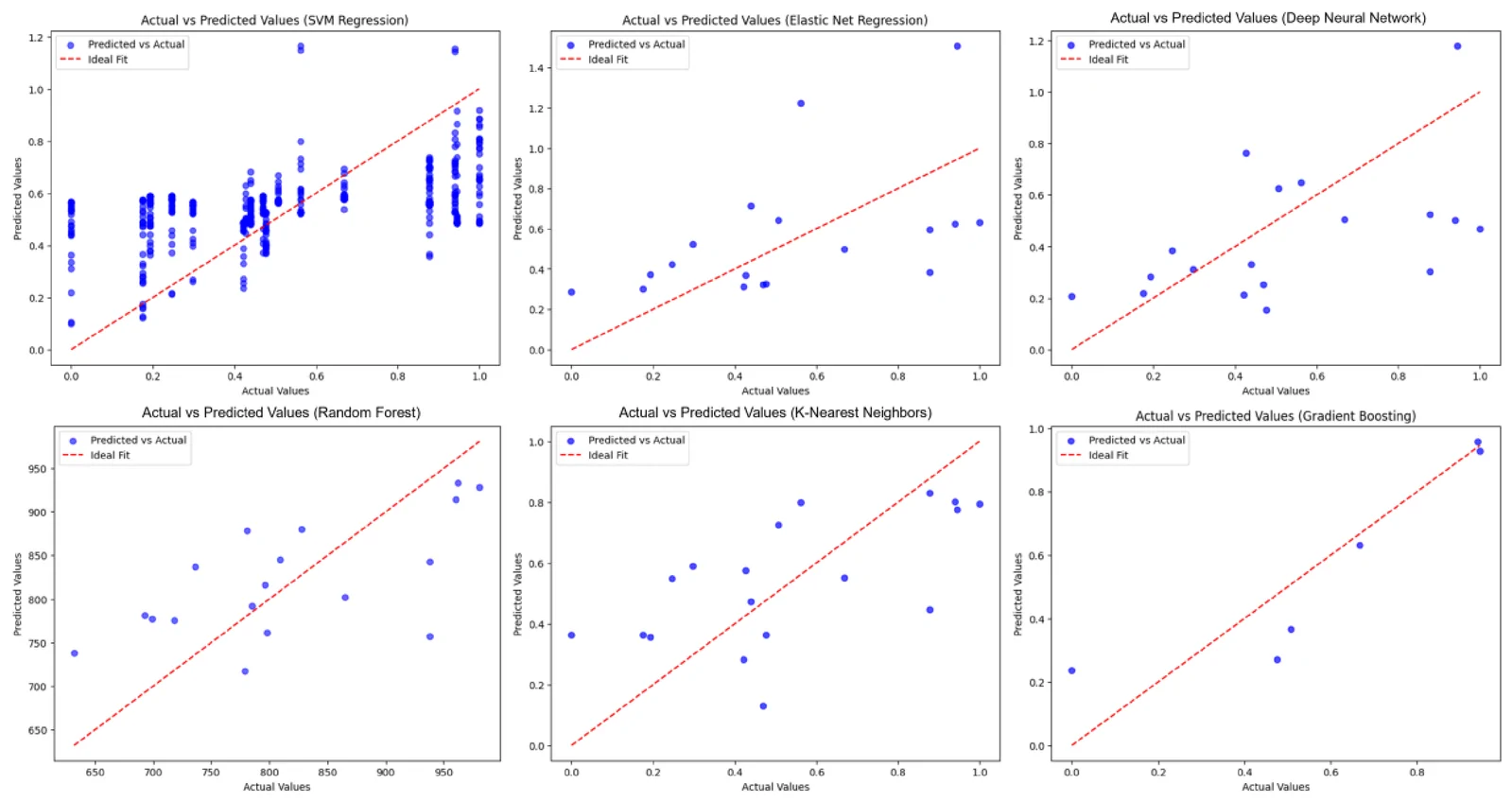
Comparison of actual and predicted values for the six regression algorithms.

**Figure 2:**
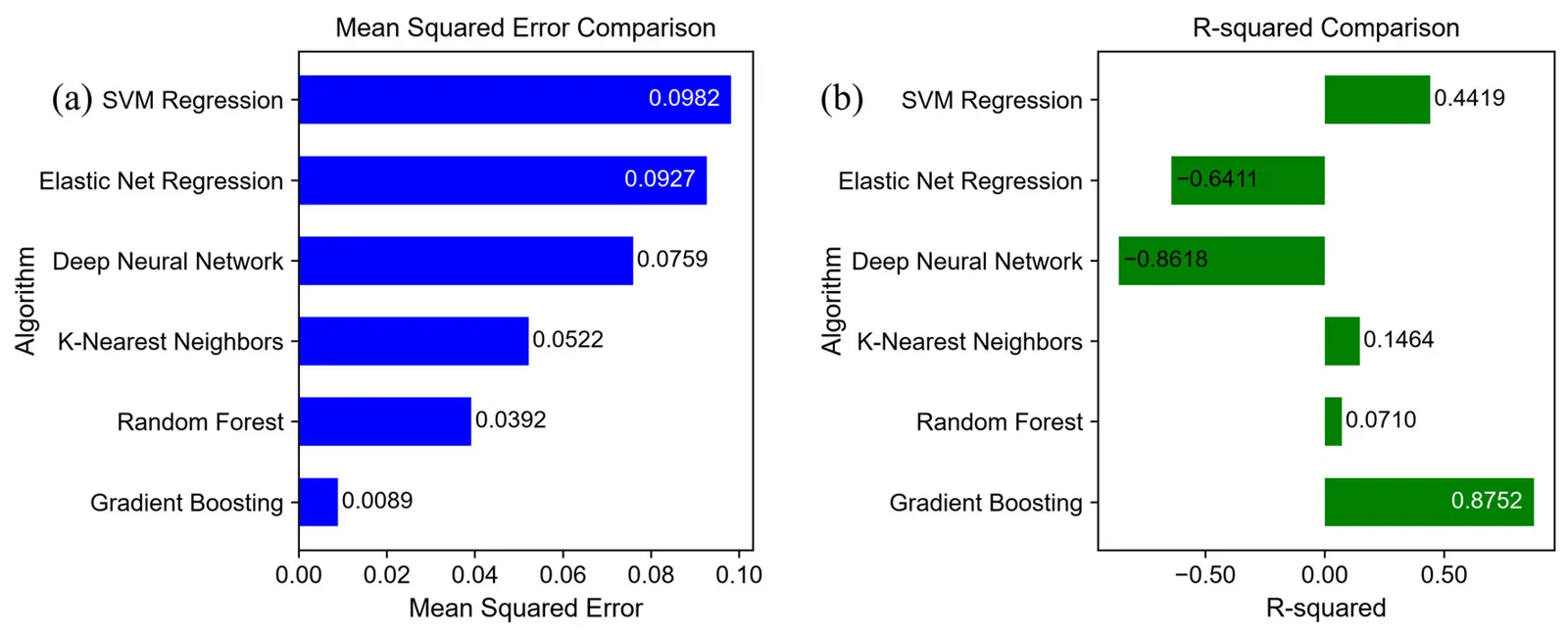
Performance comparison of six ML algorithms: (**a**) MSE; (**b**) R^2^.

Figure 1 and Figure 2 compare the test-set predictions and summary metrics for the six algorithms. GB achieved the best performance, with R^2^ = 0.8752 and MSE = 0.0089 on the normalized yield-strength response. Its feature-importance values were 0.2635 for A_1_, 0.2191 for T_1_, 0.2428 for A_2_, and 0.2746 for T_2_.

These values indicate that all four DSC descriptors contributed and that the second peak temperature had the highest feature importance, although its contribution was not dominant. The two DSC regions are therefore interpreted as informative descriptors of heating-stage reaction/phase-transformation behavior; they are not themselves predicted sintering temperatures. Given the small dataset (*n* = 30) and the sensitivity of models trained on small datasets to partitioning, the GB model is used here as an exploratory screening tool.

### 2.2. Material Preparation and Sintering

Five alloys were prepared with nominal compositions Ti-22Al-(25 − x)Nb-xRE (at.%), where x = 0, 0.5, 1.0, 1.5, and 2.0 and RE denotes the La-Ce addition introduced through an Al-La-Ce master-alloy powder (Shanghai Yunfu Nanotechnology Co., Ltd., Shanghai, China). Thus, Ti and Al were held constant while RE replaced Nb on an equal atomic-percent basis, giving Ti-22Al-25Nb, Ti-22Al-24.5Nb-0.5RE, Ti-22Al-24Nb-1.0RE, Ti-22Al-23.5Nb-1.5RE, and Ti-22Al-23Nb-2.0RE. This design avoids changing Ti and Al simultaneously, but it necessarily couples increasing RE with decreasing Nb; the consequences of this covariation are addressed explicitly in Section 3.1. Elemental Ti (Chengdu Guoheng Technology Co., Ltd., Chengdu, China) and Nb (Shanghai Yunfu Nanotechnology Co., Ltd., Shanghai, China) powders, Al-Nb master-alloy powder (Shanghai Yunfu Nanotechnology Co., Ltd., Shanghai, China), and Al-RE master-alloy powder were mechanically mixed by ball milling using an SFM-1 mill (Hefei Kejing, Hefei, China) at 800 r min^−1^ for 10 min and compacted by cold isostatic pressing at 300 MPa using a KJYu150 press (Shanxi Golden Kaiyuan Co., Ltd., Taiyuan, China). The green compacts were vacuum-sealed and sintered in a GSL-1800X tube furnace (Hefei Kejing, Hefei, China) at 1400 °C for 3 h using a heating rate of 10 °C min^−1^.

**Figure 3:**
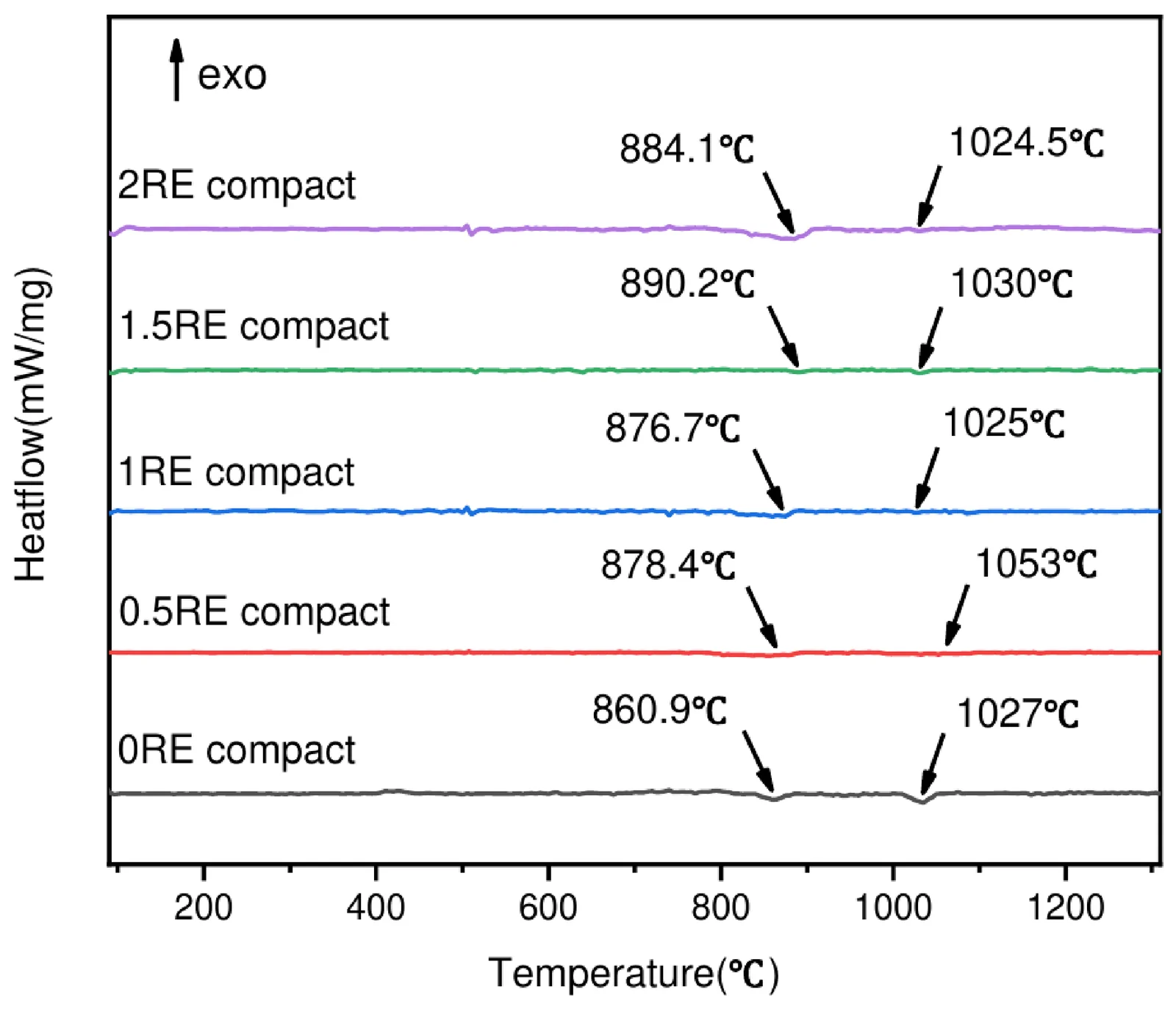
DSC curves of the five Ti-22Al-(25 − x)Nb-xRE green compacts; arrows indicate the two endothermic features used as ML descriptors.

The 1400 °C/3 h schedule was an established laboratory sintering condition selected from prior processing experience to provide sufficient diffusion and densification of the Ti-Al-Nb powder compact; it was not an output of the ML model. DSC was performed using an STA 449 F3 thermal analyzer (NETZSCH-Gerätebau GmbH, Selb, Germany) on the five green compacts during heating to 1200 °C at 10 °C min^−1^. The DSC curves are shown in Figure 3. The first peak temperatures were approximately 860.9, 878.4, 876.7, 890.2, and 884.1 °C for 0, 0.5, 1.0, 1.5, and 2.0RE, respectively, while the second peaks occurred at approximately 1027, 1053, 1024.9, 1030, and 1024.5 °C.

In our research group’s previous study of Ti/Al-Nb/Nb powder compacts, endothermic peaks at 907 and 1055 °C were correlated by quenched-state XRD with α-Ti → β-Ti and α_2_ → B2 transformations, respectively [28]. On that experimental basis, the lower- and higher-temperature peak groups in the present five alloys are assigned, respectively, to α-Ti → β-Ti and α_2_ → B2 transformation ranges. The peak shifts are consistent with composition-dependent transformation temperatures, but the concurrent Nb decrease and RE addition prevent attribution to RE alone. These DSC features are therefore used as heating-stage transformation descriptors rather than as direct predictions of the final sintering temperature.

**Figure 4:**
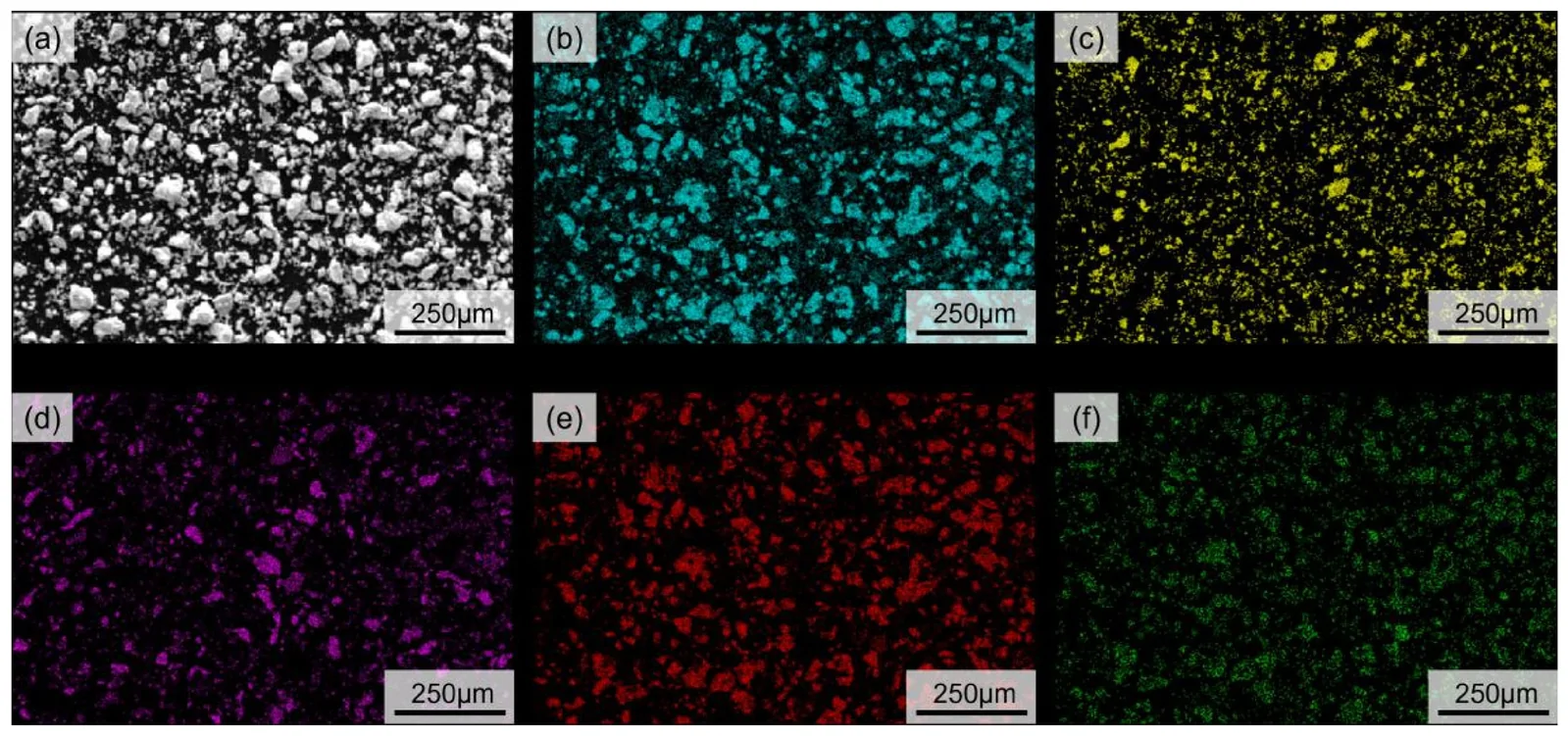
SEM image and EDS elemental mappings of the ball-milled mixed powder: (**a**) SEM image; (**b**) Ti; (**c**) Al; (**d**) Nb; (**e**) La; (**f**) Ce.

Figure 4 shows the SEM image and EDS elemental maps of the ball-milled mixed powder. The maps show the spatial distributions of Ti, Al, Nb, La, and Ce in the examined powder field. The mixture comprises elemental Ti and Nb powders and Al–Nb and Al–La–Ce master-alloy powders. The SEM image shows irregularly shaped particles with varying sizes (Figure 4a). The Ti map reflects the distribution of the elemental Ti powder, whereas Nb originates from both elemental Nb and Al–Nb powders (Figure 4b,d). Al is supplied by both Al–Nb and Al–La–Ce powders (Figure 4c), while the dispersed La and Ce signals indicate the distribution of the Al–La–Ce addition (Figure 4e,f). These elemental distributions are consistent with intermixing of the four feedstocks, while reflecting the distinct compositions of the constituent powders.

### 2.3. Characterization and Testing

The constituent phases were identified by X-ray diffraction (XRD; SmartLab, Rigaku, Tokyo, Japan), and their fractions were quantified by Rietveld refinement. Microstructures, phase morphology, particle distribution, and fracture surfaces were examined by scanning electron microscopy (SEM; MIRA3, TESCAN, Brno, Czech Republic), with energy-dispersive spectroscopy (EDS) used for qualitative elemental identification. EDS measurements from grain-boundary α_2_ regions and B2-matrix regions were summarized only as semi-quantitative apparent oxygen contents for within-series comparison; conventional SEM-EDS cannot provide reliable bulk or exact phase-resolved oxygen concentrations. Grain boundaries were manually delineated in ImageJ (version 1.54g) and equivalent-circle diameters were calculated. More than 500 grains were measured for each alloy. RE_2_O_3_ particles were measured by the same manual outlining and equivalent-circle diameter procedure. O-phase lath widths were measured perpendicular to their length using the ImageJ line tool. Porosity and O-phase width measurements were included in the analysis for each composition. Error bars represent sample standard deviations. No EBSD, TEM, or SAED measurements were available; phase assignments in SEM images are therefore based on XRD/Rietveld results together with contrast, morphology, and EDS chemistry rather than independent crystallographic mapping.

**Figure 5:**
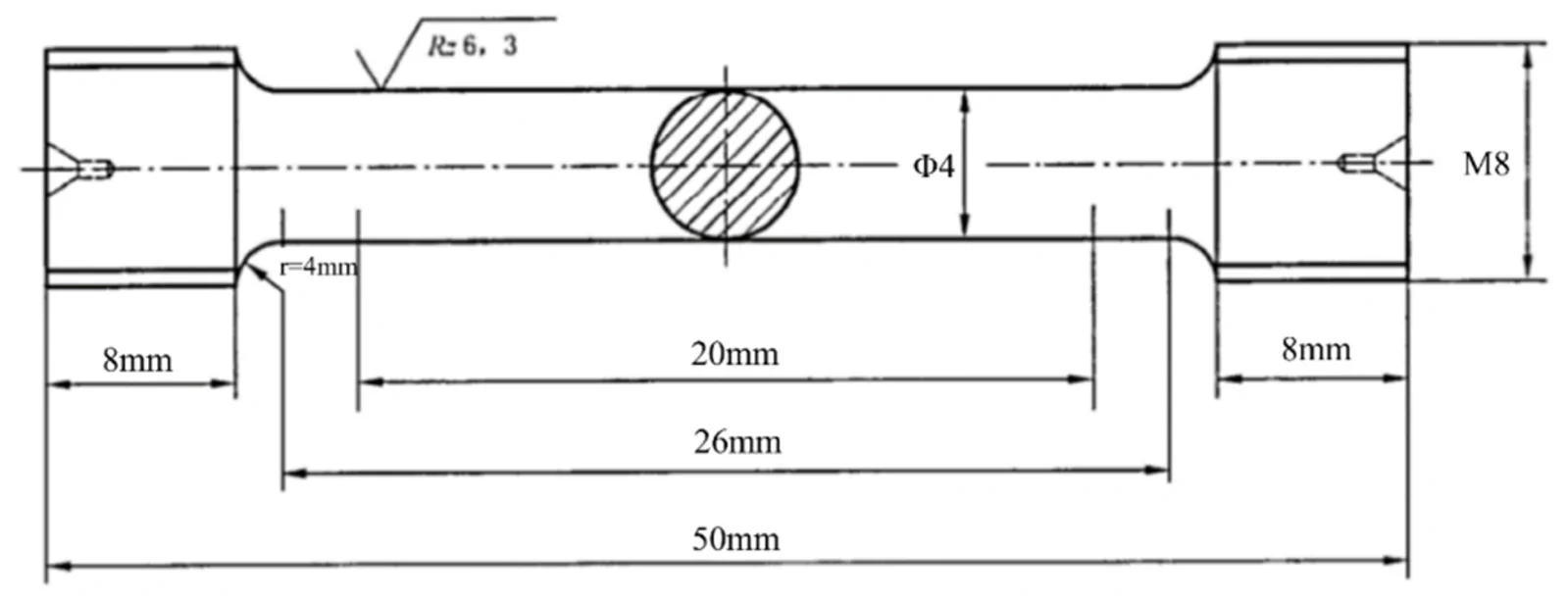
Geometry and dimensions of the miniature threaded tensile specimen used in this study.

Uniaxial tensile tests were performed at 25 °C and 600 °C using an electronic universal testing machine (5985, Instron, Norwood, MA, USA) at a nominal strain rate of 1 × 10^−3^ s^−1^. The miniature threaded cylindrical specimens had a 20 mm gauge length, a 4 mm gauge diameter, a 26 mm parallel section, an overall length of 50 mm, and M8 threaded ends (Figure 5). Three specimens of each composition were tested at each temperature. Only one complete tensile dataset is available for each combination of composition and temperature, so the curves shown below are representative and the variability in strength and true fracture strain cannot be reliably assessed; error bars are therefore not reported.

True stress–strain curves are reported in this work. True stress is defined as *σ*_t_ = P/A, where A is the instantaneous cross-sectional area. The peak true stress, *σ*_t,max_, and true fracture strain, *ε*_t,f_, are read from these curves. Fracture-surface SEM observations were used to relate mechanical response to fracture morphology and RE-containing oxide particles.

## 3. Results and Discussion

### 3.1. Phase Composition and Crystal Structure

**Figure 6:**
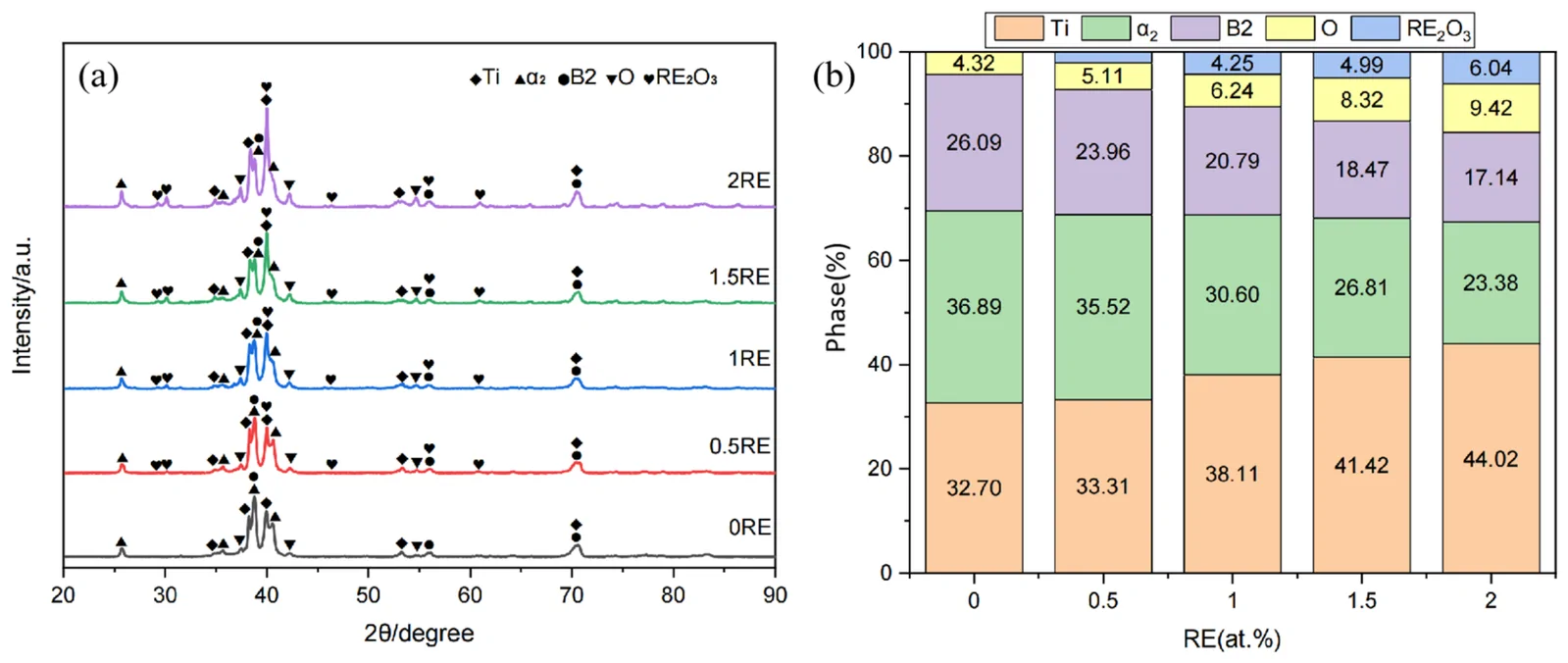
(**a**) XRD patterns of Ti-22Al-(25 − x)Nb-xRE alloys; (**b**) Rietveld-refined phase fractions as a function of RE content.

The XRD patterns and Rietveld results (Figure 6) show that the RE-free alloy contains α-Ti, B2, α_2_, and O phases, while RE-containing alloys additionally exhibit rare-earth-oxide reflections. The peaks labeled “Ti” in the original XRD plot are assigned here to α-Ti, the hexagonal close-packed Ti-rich solid solution (P6_3_/mmc). The α_2_ phase is ordered Ti_3_Al with the hexagonal D0_19_ structure (P6_3_/mmc), and is mainly located at grain boundaries; the O phase is orthorhombic Ti_2_AlNb (Cmcm) and appears as intragranular needles or laths. RE_2_O_3_ in Figure 6a denotes a mixture of La_2_O_3_ and Ce_2_O_3_, collectively labeled because their closely spaced diffraction peaks cannot be distinguished separately. The crystal structures of both oxides are listed in Table 2. Phase identification is based on XRD/Rietveld refinement and is supported, where possible, by SEM/EDS observations [5,14,15,29,30].

The phase fractions change systematically across the designed series. The α_2_ fraction decreases from 36.89% at 0RE to 23.38% at 2.0RE, and the B2 fraction decreases from 26.09% to 17.14%. In contrast, the O phase increases from 4.32% to 9.42%, while the RE_2_O_3_-type fraction rises from zero to 6.04%; the α-Ti fraction increases from 32.70% to 44.02%. These trends should not be attributed to RE alone, because Nb simultaneously decreases from 25 to 23 at.%. Nb is a β/B2-stabilizing element, so the decrease in Nb can itself alter β/B2 stability and transformation behavior. The observed phase evolution is therefore interpreted as a combined consequence of RE addition, RE–O interactions, and the 2 at.% reduction in Nb, rather than as an isolated RE-only effect.

**Figure 7:**
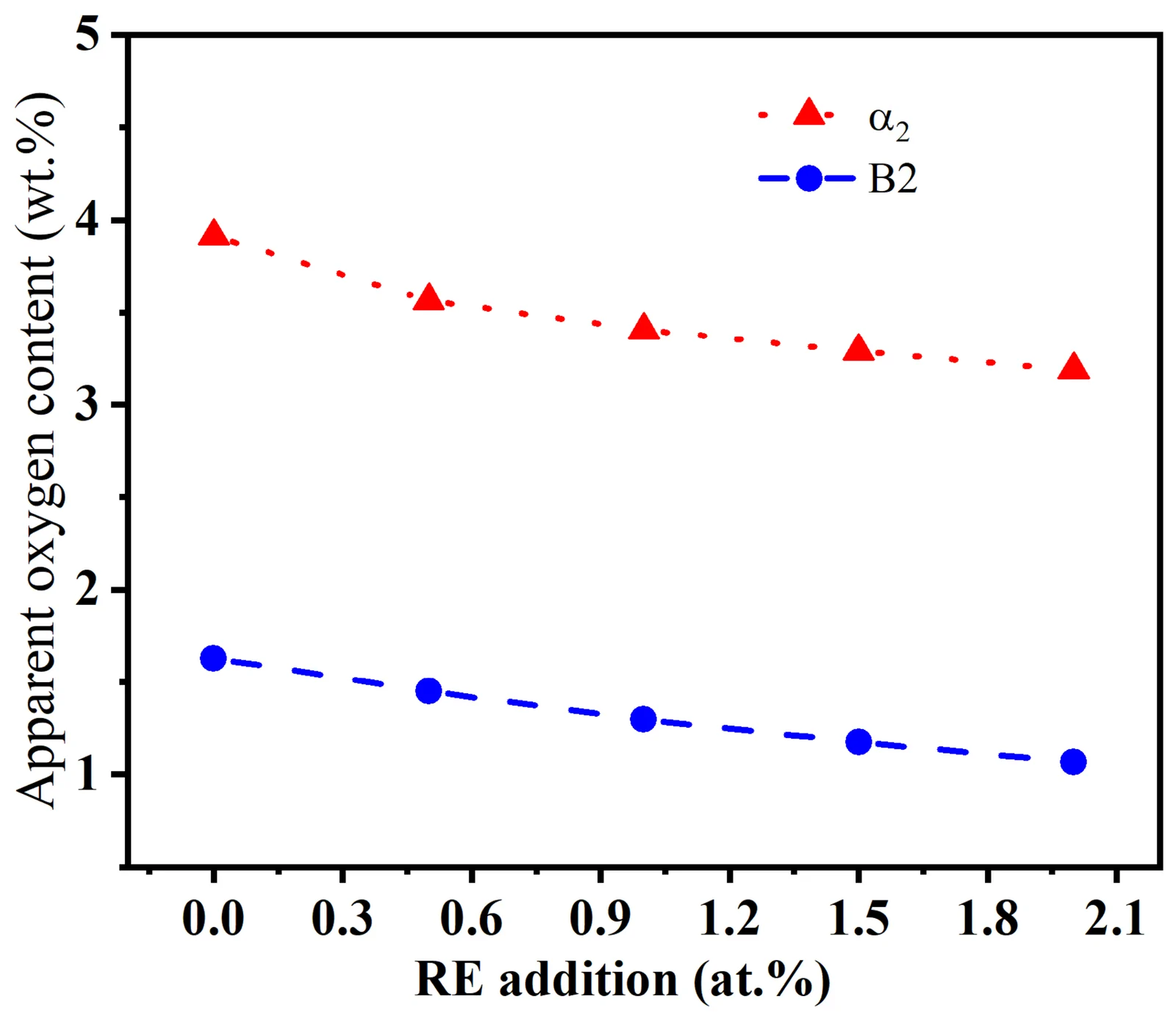
EDS-derived apparent oxygen contents in α_2_ and B2-matrix regions of Ti-22Al-(25 − x)Nb-xRE alloys. Values from conventional SEM-EDS are used only for within-series trend comparison; they are not accurate bulk or phase-resolved oxygen contents, and the number of replicate measurements and the measurement uncertainty are unavailable.

The emergence of RE_2_O_3_-type reflections indicates the formation of stable RE-containing oxide products. The SEM-EDS dataset shows that the apparent oxygen content decreases monotonically from 3.913 to 3.188 wt.% in grain-boundary α_2_ regions and from 1.630 to 1.065 wt.% in B2-matrix regions as the RE addition increases from 0 to 2.0 at.% (Figure 7).

This within-series trend is qualitatively consistent with less oxygen remaining in the examined metallic regions as RE-containing oxides form. Previous work on YH_2_-added PM Ti-Nb-Ta alloys showed through quantitative analysis that Y_2_O_3_ formation can reduce solid-solution oxygen in metallic phases and alter oxygen partitioning [23]. In the present alloys, however, the SEM-EDS values are semi-quantitative and the number of replicate measurements and the measurement uncertainty are unavailable. The XRD/SEM/EDS observations are therefore consistent with, but do not directly prove, quantitative oxygen transfer or matrix deoxygenation in the La-Ce-containing system. The phase-fraction changes may reflect the combined effects of oxygen partitioning and the simultaneous reduction in Nb content.

### 3.2. Microstructure Evolution and the RE-O Mechanism

**Figure 8:**
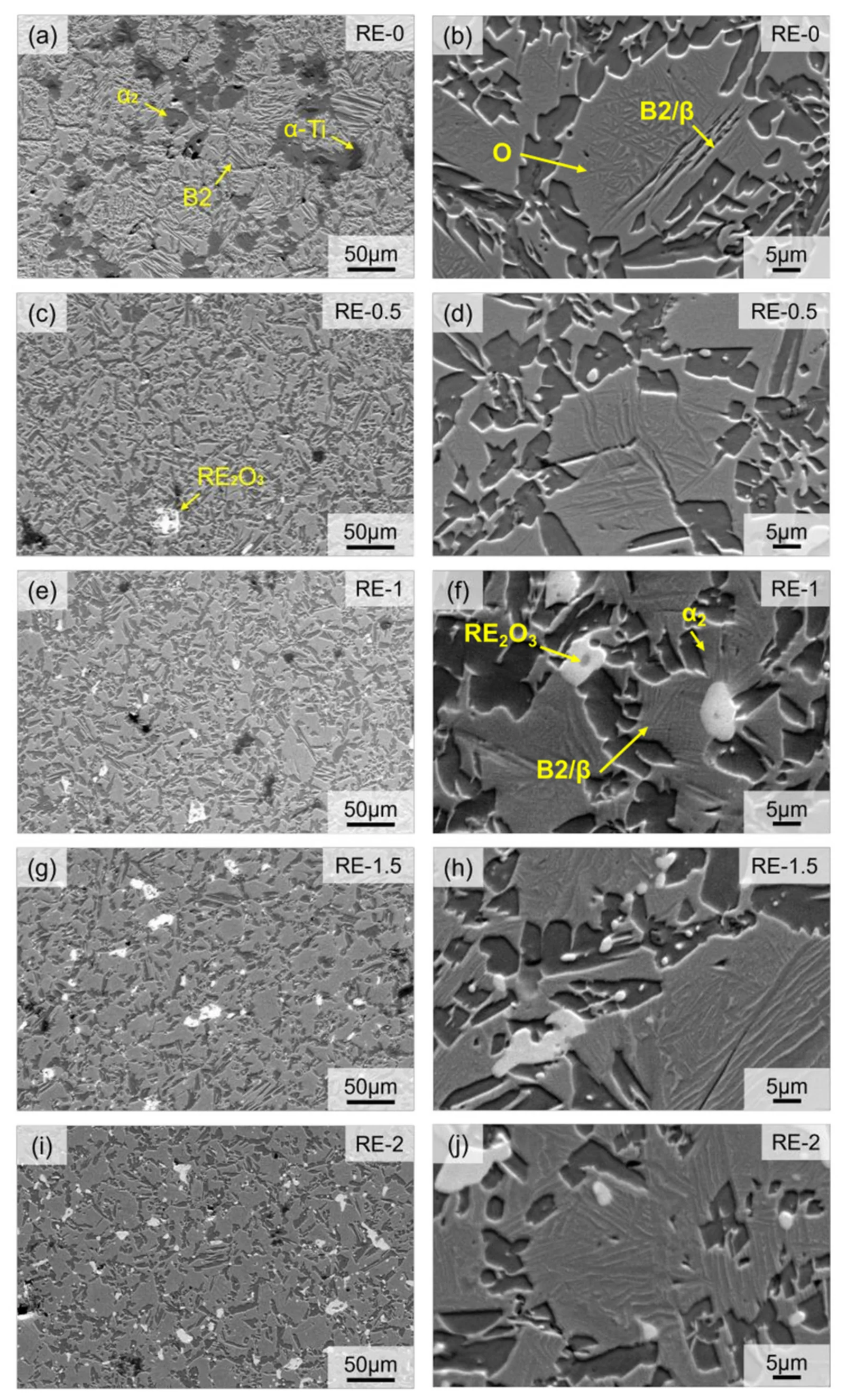
SEM images of Ti-22Al-(25 − x)Nb-xRE alloys: (**a**,**b**) 0RE; (**c**,**d**) 0.5RE; (**e**,**f**) 1.0RE; (**g**,**h**) 1.5RE; (**i**,**j**) 2.0RE. The brightest particles are RE_2_O_3_-type oxides; the light-gray continuous phase is the B2 matrix; intermediate-gray intragranular needles are assigned to O phase; and the dark gray grain-boundary constituent is α_2_. The “α-Ti” label in panel (**a**) denotes the hcp Ti-rich solid solution; B2/β labels denote bcc-related matrix regions.

Figure 8 shows the SEM microstructures of the five alloys. The light-gray continuous regions constitute the B2 matrix; the dark gray, predominantly grain-boundary constituent is assigned to α_2_; the intermediate-gray intragranular acicular/lath features are the O phase; and the brightest particles in the RE-containing alloys are RE_2_O_3_-type oxides. The α-Ti phase is hcp, whereas β-Ti and B2 are disordered and ordered bcc phases, respectively. The B2/β labels denote bcc-related matrix regions without resolving their ordering state.

**Figure 9:**
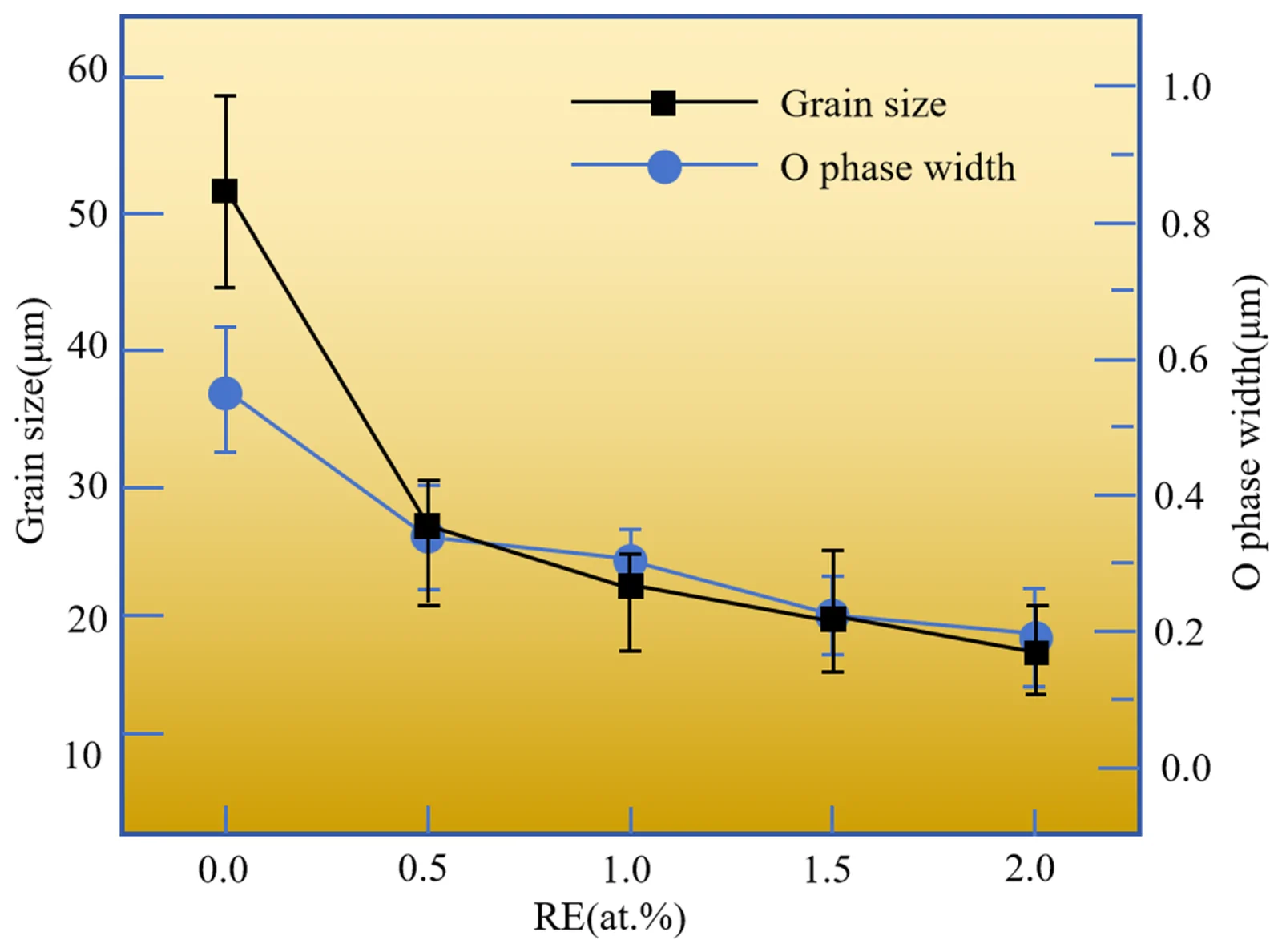
Variation in mean grain size and O-phase width with RE content in Ti-22Al-(25 − x)Nb-xRE alloys. Grain size was determined from more than 500 manually outlined grains per alloy using ImageJ; equivalent-circle diameters were calculated from the outlined areas. O-phase lath widths were measured perpendicular to their length using the ImageJ line tool.

Figure 9 summarizes the mean grain size and O-phase width as a function of RE content. Increasing RE substitution is accompanied by microstructural refinement. ImageJ analysis of more than 500 manually delineated grains per alloy gave mean grain sizes of 51.6 ± 5.4, 27.0 ± 4.6, 22.8 ± 3.9, 20.4 ± 3.2, and 18.1 ± 2.5 μm for 0, 0.5, 1.0, 1.5, and 2.0RE, respectively; the error bars are the sample standard deviations of the results from five image fields per composition. The corresponding porosity values are 0.292 ± 0.043%, 0.102 ± 0.022%, 0.0766 ± 0.0183%, 0.0617 ± 0.0144%, and 0.0864 ± 0.0195%, confirming a minimum at 1.5RE followed by an increase at 2.0RE. The RE_2_O_3_ particle measurements yield mean diameters of 10.19 ± 3.20 μm, 9.75 ± 2.66 μm, 6.21 ± 2.69 μm, and 5.04 ± 2.36 μm for 0.5, 1.0, 1.5, and 2.0RE, respectively. These particles are micrometer-sized. RE-rich oxide particles observed at or near grain/interphase boundaries may contribute to refinement [23,31,32,33,34,35], but a quantitative Zener-pinning calculation cannot be made from the available data.

The monotonic grain-size reduction (51.6 to 18.1 μm, 64.9%) is therefore treated as an experimentally observed correlation with RE substitution, while the individual contributions of oxide pinning, densification, phase-fraction changes, and the simultaneous Nb decrease cannot be isolated from the present five-composition design. Dehydrogenation-assisted sintering [36] and hot isostatic pressing [37] further illustrate the sensitivity of titanium densification and microstructure to processing. This distinction is important because the compositional series was designed for practical alloy screening rather than as a fully orthogonal experiment separating RE and Nb effects.

The measured O-phase widths (mean ± sample standard deviation) decrease from 0.550 ± 0.191 μm in the RE-free alloy to 0.338 ± 0.084 μm, 0.304 ± 0.048 μm, 0.222 ± 0.097 μm, and 0.194 ± 0.080 μm in the 0.5, 1.0, 1.5, and 2.0RE alloys, respectively. Rather than assigning this trend solely to oxygen redistribution through RE-containing oxide formation, the result is interpreted as evidence that the combined changes in oxygen partitioning, phase fractions, and alloy composition alter O-phase precipitation/coarsening during cooling. A finer O/B2 morphology can increase interface density and shorten characteristic slip distances, which may contribute to the improved balance of strength and deformation compatibility [38,39,40]. Related microstructure–property relationships have been demonstrated through ω precipitation in Ti–Mo–Al–Nb [41], intermetallic morphology in Ti–Nb–Al–Cu–Ni [42], and bimorphic or hierarchical α/β structures in titanium alloys [43,44].

The present results suggest three coupled contributions to the RE-O mechanism: formation of stable RE-containing oxides accompanied by a decreasing semi-quantitative EDS oxygen trend in the examined grain-boundary α_2_ and B2-matrix regions; grain-boundary pinning by micrometer-scale RE_2_O_3_ particles; and modification of the α-Ti/α_2_/B2/O transformation path and the resulting O-phase morphology. Oxide formation, the EDS trend, and microstructural refinement support this interpretation, while the detailed oxygen-transfer pathway and accurate phase-specific oxygen concentrations remain to be verified by quantitative oxygen analysis and higher-resolution characterization.

The proposed mechanism can be summarized as follows:

Oxygen redistribution: RE reacts readily with oxygen to form stable RE-containing oxides. Together with the decreasing EDS-derived apparent oxygen contents in the examined metallic regions, the quantitatively verified RE-assisted oxygen redistribution reported for other PM Ti alloys [23] supports the possibility of a similar local reduction in solid-solution oxygen here. The magnitude of that effect cannot be established from the present SEM-EDS data.

Particle/grain-boundary pinning: RE_2_O_3_-type particles are frequently located at or near grain boundaries. Their presence is consistent with impeded grain-boundary migration and with the observed grain refinement, although number-density data are unavailable for a full quantitative pinning analysis.

Transformation/morphology control: changes in α-Ti, α_2_, B2, and O fractions and the decrease in O-lath width indicate modified transformation behavior. Because Nb decreases as RE increases, this effect should be interpreted as a coupled compositional and oxygen-partitioning response rather than an RE-only transformation effect. Composition-dependent phase evolution is also reported for SPS-processed Ti–24Nb–xZr alloys [45].

### 3.3. Mechanical Properties

Representative true stress–strain curves were obtained at 25 °C and 600 °C. Although three specimens were tested per condition (Section 2.3), only one complete curve per condition is available. The results are therefore presented without standard deviations, and test repeatability cannot be reliably assessed.

**Figure 10:**
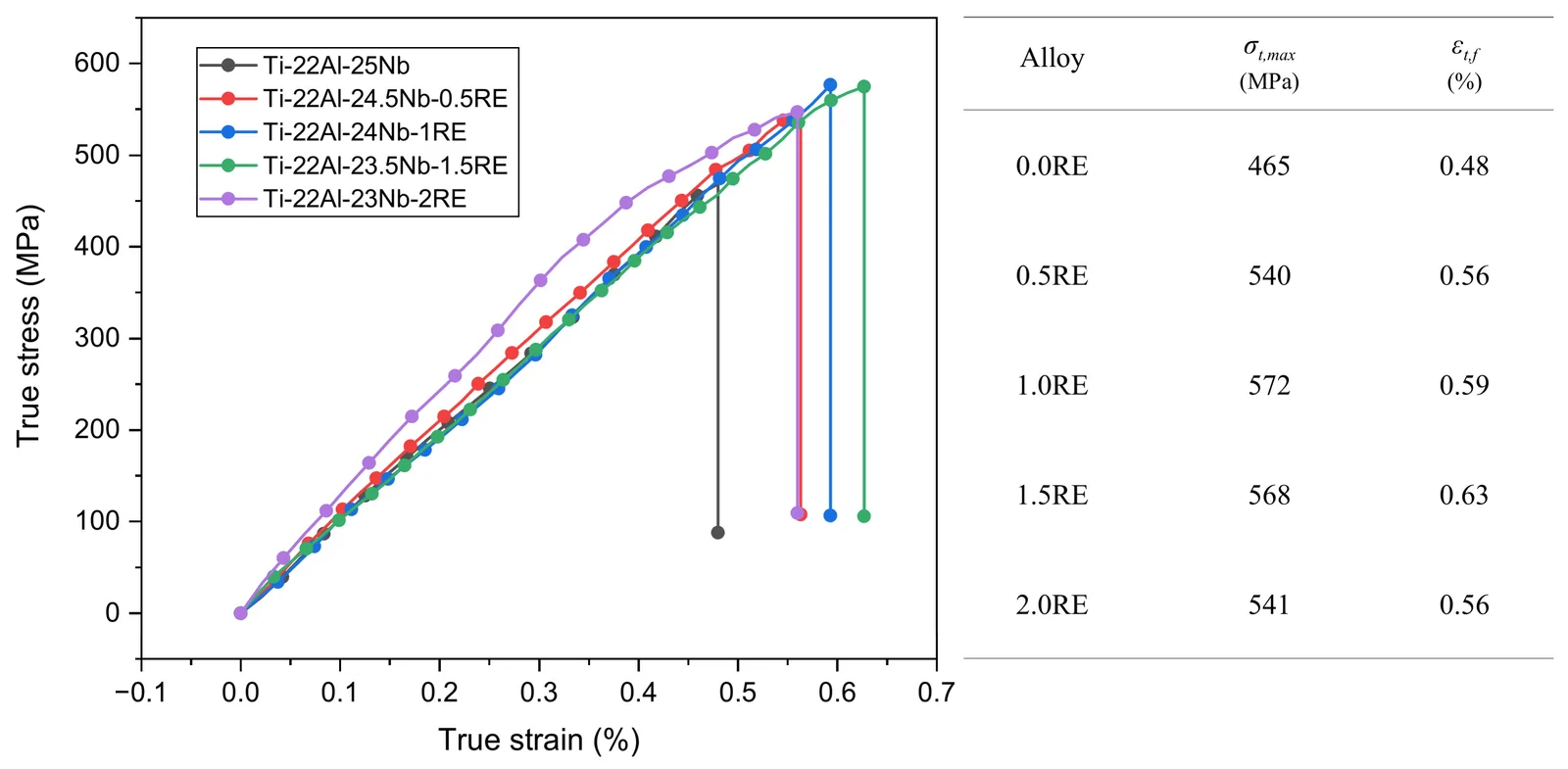
Representative true stress–strain curves of Ti-22Al-(25 − x)Nb-xRE alloys at room temperature. σ_t,max_: peak true stress; ε_t,f_: true fracture strain.

Figure 10 shows the room-temperature true stress–strain curves. All five alloys fracture at very small strains and therefore remain predominantly brittle, but RE substitution changes both *σ*_t,max_ and true fracture strain. The RE-free alloy gives 465 MPa and 0.48% true fracture strain. The 0.5RE alloy reaches 540 MPa and 0.56%; 1.0RE reaches the maximum room-temperature *σ*_t,max_ of 572 MPa with 0.59% true fracture strain; 1.5RE retains a similar *σ*_t,max_ of 568 MPa while giving the maximum true fracture strain of 0.63%; and the values for 2.0RE decrease to 541 MPa and 0.56%.

Thus, room-temperature strength and ductility do not share the same optimal composition. The 1.0RE composition maximizes *σ*_t,max_, whereas 1.5RE maximizes true fracture strain and retains nearly the same strength. The 30.8% increase in true fracture strain from 0.48% to 0.63% coincides with reduced porosity, a finer grain size, less continuous coarse α_2_ at grain boundaries, and a refined O/B2 morphology. These correlations are consistent with improved deformation compatibility, but they do not by themselves prove a unique strengthening mechanism.

The strength increase from 0RE to 1.0–1.5RE is compatible with Hall–Petch-type grain refinement and particle strengthening [34,35,46], while the subsequent decline at 2.0RE coincides with a rise in porosity and a less favorable balance of particle/matrix interfaces. Because the mean RE_2_O_3_ particle diameters are approximately 5–10 μm and particle number density and area/volume fraction are unavailable, a dispersion-strengthening relationship cannot be quantitatively established. In addition, the concurrent 25 → 23 at.% Nb change may contribute to the phase-balance and mechanical-property trends and cannot be fully separated from the RE effect in the present design. Other titanium systems show contributions from texture-dependent yielding [47] and ceramic reinforcement [48], underscoring the need to distinguish individual strengthening mechanisms.

The mechanical response is therefore interpreted from the combined evidence: grain refinement, reduced porosity up to 1.5RE, reduced continuity of coarse α_2_ regions, modified O/B2 morphology, and the presence of RE-rich oxide particles. The 2.0RE composition shows that further RE substitution is not continuously beneficial, because porosity rises and both strength and true fracture strain fall from their intermediate-composition maxima.

**Figure 11:**
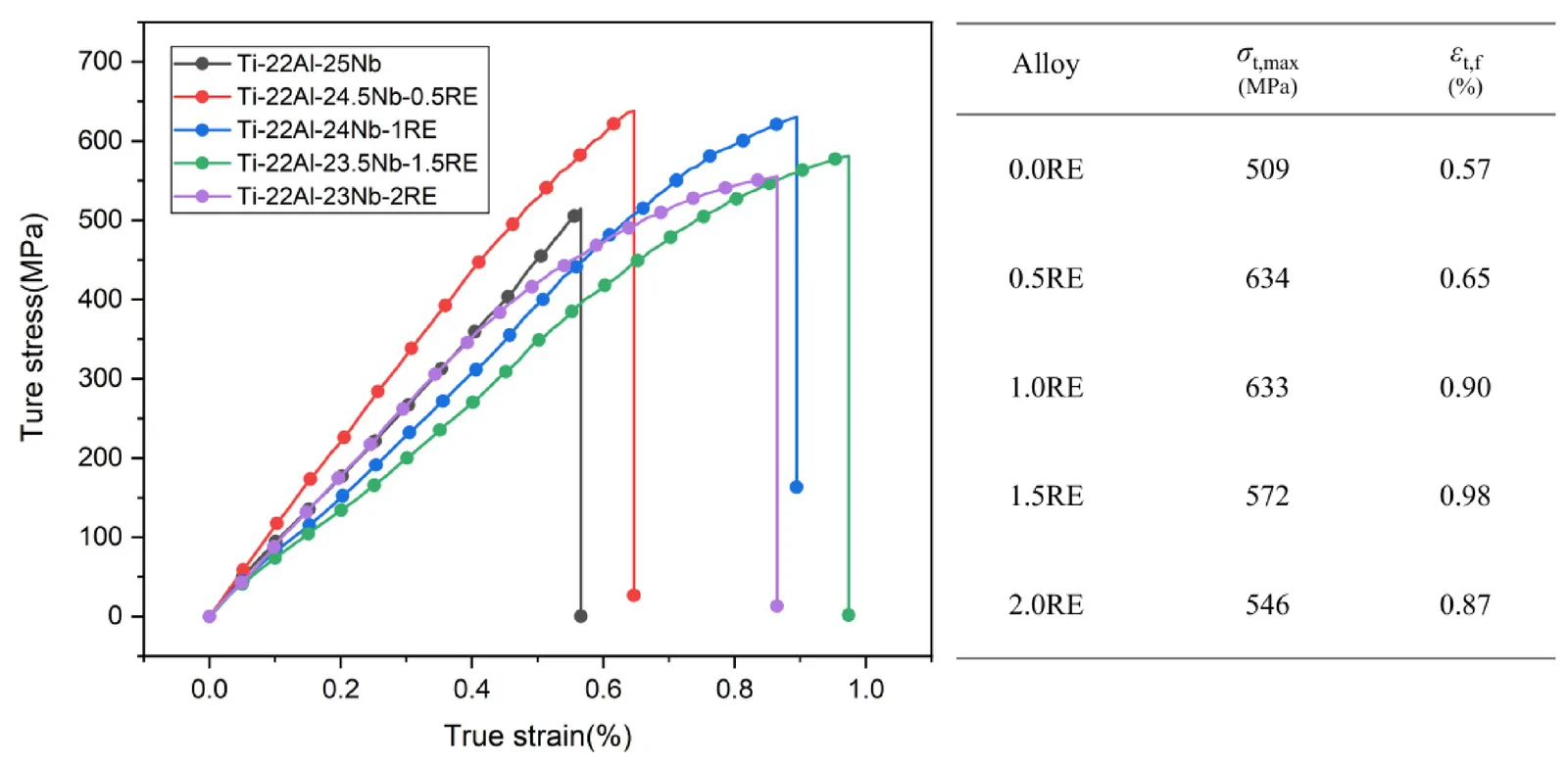
Representative true stress–strain curves of Ti-22Al-(25 − x)Nb-xRE alloys at 600 °C. σ_t,max_: peak true stress; ε_t,f_: true fracture strain.

Figure 11 presents the 600 °C true stress–strain curves. Relative to room temperature, the alloys show greater strain before fracture. The RE-free alloy reaches 509 MPa and 0.57% true fracture strain. The 0.5RE and 1.0RE alloys provide the highest elevated-temperature strengths, 634 and 633 MPa, with true fracture strains of 0.65% and 0.90%, respectively. The 1.5RE alloy has a lower *σ*_t,max_ of 572 MPa but reaches the maximum true fracture strain of 0.98%, while the corresponding values for the 2.0RE alloy decrease to 546 MPa and 0.87%.

The 600 °C results reinforce the need to define “optimal” composition precisely. The highest elevated-temperature *σ*_t,max_ occurs at 0.5RE (634 MPa), essentially matched by 1.0RE (633 MPa), whereas the highest true fracture strain occurs at 1.5RE (0.98%, 71.9% above the 0RE value of 0.57%). Thus, 1.5RE is not the strength optimum at 600 °C; it is selected as the best overall composition only when room-temperature strength retention, maximum room-temperature true fracture strain, maximum 600 °C true fracture strain, low porosity, and microstructural uniformity are considered together.

The elevated-temperature strength retained by the RE-containing alloys is consistent with the thermal stability of RE oxide particles and their potential to hinder boundary/dislocation motion [23]. At the same time, excessive refinement or particle/porosity changes can reduce grain-boundary cohesion or promote boundary-mediated deformation at elevated temperature. These competing effects provide a plausible explanation for the non-monotonic strength trend, but direct dislocation-density measurements were not performed in the present study.

Across both temperatures, the practical composition choice is therefore a balance of multiple properties rather than the maximum of a single property. On that basis, 1.5 at.% RE is considered to provide the best overall balance in this five-composition series: it retains a peak true stress of 568 MPa while maximizing room-temperature true fracture strain (0.63%); it also maximizes at 600 °C the true fracture strain (0.98%). The term “optimal” is used in this restricted sense throughout this study.

### 3.4. Fracture Mechanism Analysis

**Figure 12:**
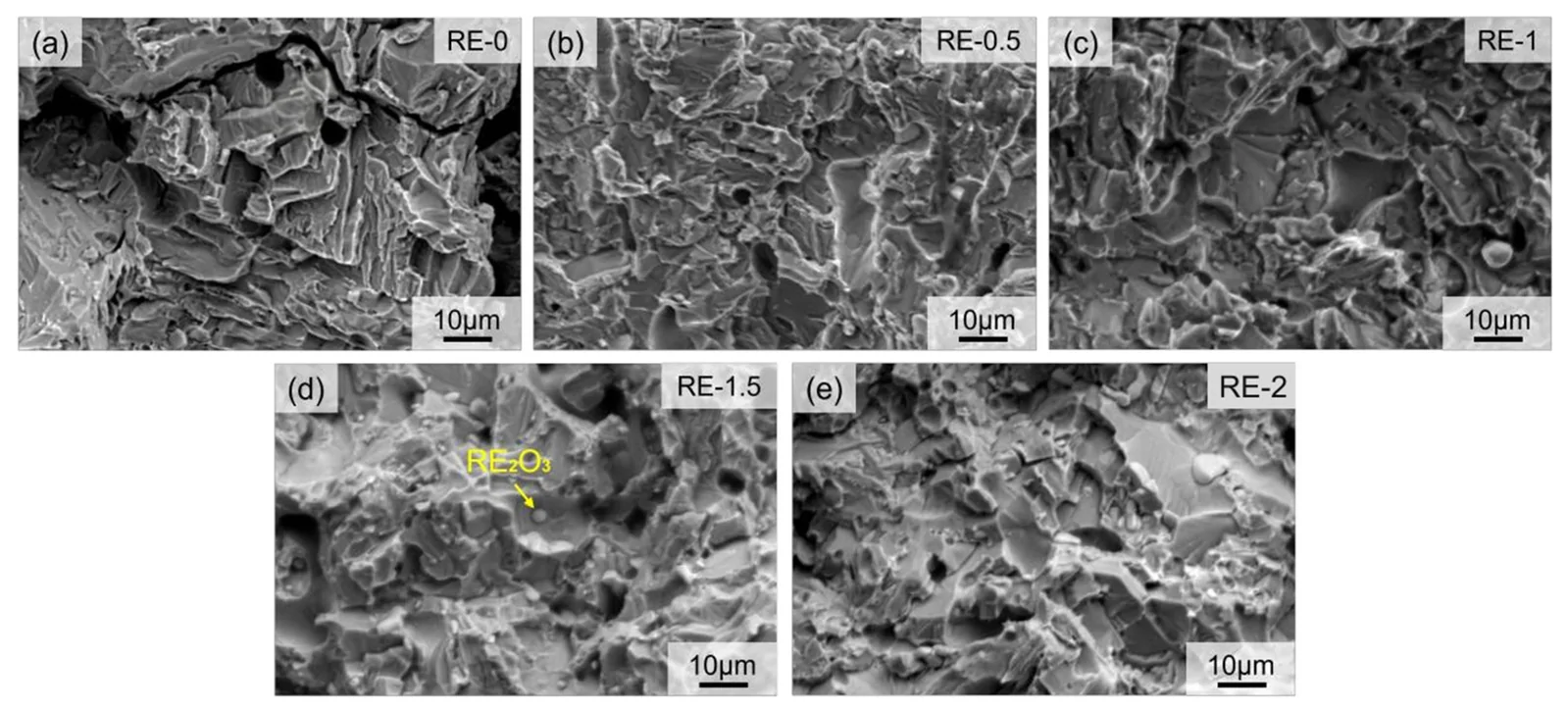
SEM images of room-temperature tensile fracture surfaces of Ti-22Al-(25 − x)Nb-xRE alloys: (**a**) 0RE; (**b**) 0.5RE; (**c**) 1.0RE; (**d**) 1.5RE; (**e**) 2.0RE.

Figure 12 shows the room-temperature tensile fracture surfaces. The RE-free alloy exhibits predominantly cleavage-like morphology and cracking associated with coarse α_2_/B2 interfacial regions. With RE substitution, localized dimples and more tortuous fracture paths become more apparent, while bright RE-rich particles are observed in some dimpled regions. These observations are consistent with the improved true fracture strain but do not, by themselves, demonstrate a unique crack-deflection or energy-dissipation mechanism.

The 1.5RE alloy combines the lowest measured porosity (≈0.062%) with the highest room-temperature true fracture strain. Reducing internal voids decreases the density of obvious crack-initiation sites, while refinement of the coarse grain-boundary α_2_ morphology may reduce the continuity of weak paths. At 2.0RE, the porosity rises to ≈0.086% and the mechanical properties decline, consistent with a less favorable defect/interface population.

Overall, room-temperature fracture remains predominantly brittle across the entire composition series because the total true fracture strain is below 1%. The appearance of local dimples in RE-containing alloys should therefore be interpreted as a relative increase in ductile microvoid activity within an otherwise brittle-dominated fracture process, rather than a complete transition to ductile fracture.

The fracture observations support the same multi-factor interpretation developed from the phase and microstructure results: moderate RE substitution is associated with lower porosity, finer grains, modified α_2_/O/B2 morphology, and RE-rich oxide formation, whereas excessive substitution reduces these benefits. Because Nb changes simultaneously, the mechanical contribution of RE cannot be completely isolated without a future control series in which Nb is independently varied.

**Figure 13:**
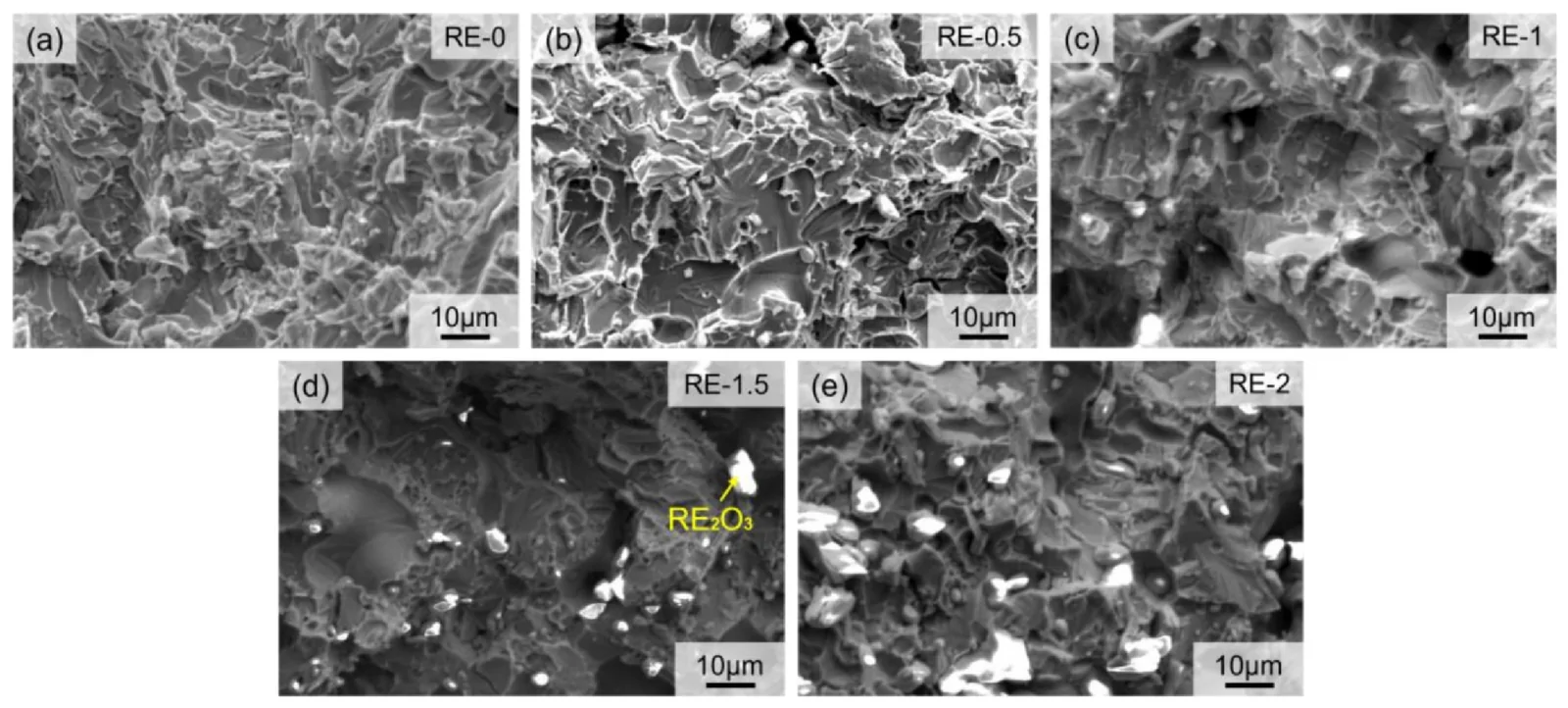
SEM images of 600 °C tensile fracture surfaces of Ti-22Al-(25 − x)Nb-xRE alloys: (**a**) 0RE; (**b**) 0.5RE; (**c**) 1.0RE; (**d**) 1.5RE; (**e**) 2.0RE.

Figure 13 shows the corresponding fracture surfaces after testing at 600 °C. Compared with room temperature, the surfaces contain more dimpled regions and less extensive cleavage-like morphology, consistent with the higher deformation capacity at elevated temperature. The 1.5RE alloy exhibits the most pronounced and relatively uniform dimple morphology among the five compositions, in agreement with its maximum 600 °C true fracture strain of 0.98%.

Bright RE-rich particles are again present within or adjacent to some dimpled regions. Such particles can act as local microvoid nucleation sites and may also impede local slip or alter crack paths, as reported for RE-oxide-containing Ti alloys [23]. In the present study, however, these roles are inferred from fracture morphology and prior literature; they were not quantified by in situ fracture observation or dislocation analysis.

### 3.5. Scope and Limitations

The following limitations should be considered when interpreting the results. First, the ML dataset contains only 30 records and the initial data shuffle used no fixed random seed, so the test-set performance should not be generalized beyond the present composition/process domain. Second, RE substitution is coupled to a 25 → 23 at.% decrease in Nb, preventing strict separation of RE and Nb effects. Third, conventional SEM-EDS provides a semi-quantitative within-series oxygen trend but does not yield reliable absolute bulk or phase-resolved oxygen concentrations; the number of replicate EDS measurements and the measurement uncertainty are unavailable, and EBSD/TEM/SAED characterization was not available. Grain size and RE_2_O_3_ particle size were measured by manual outlining in ImageJ and calculation of equivalent-circle diameters, with more than 500 grains evaluated per alloy; however, details of field selection and analyzed area, a full particle-size distribution, particle number density, and particle area/volume fraction are unavailable. Fourth, three tensile specimens of each composition were tested at each temperature, but only one complete tensile dataset is available for each combination of composition and temperature, preventing reliable assessment of variability in mechanical properties. Future work should include control alloys with independently varied Nb, quantitative oxygen analysis, high-resolution phase mapping, quantitative particle-density measurements, complete raw data from replicate tests, and a larger ML dataset. The mechanistic discussion and the definition of the best overall 1.5RE composition are therefore limited to what the present evidence supports.

## 4. Conclusions

This study combined an exploratory ML analysis with an experimental investigation of PM Ti-22Al-(25 − x)Nb-xRE alloys. The main findings are summarized below.

1. The ML dataset contained 30 in-house records described by four DSC features (A_1_, T_1_, A_2_, T_2_) and room-temperature yield strength. Six algorithms were compared using a 70/30 train–test partition and five-fold cross-validation for hyperparameter selection. The GB model achieved the best test-set performance (R^2^ = 0.8752; MSE on the normalized response = 0.0089). The ML model identified informative thermal descriptors but did not predict or optimize the 1400 °C/3 h sintering schedule, which was an established processing condition.
2. Across the Ti-22Al-(25 − x)Nb-xRE series, the mean grain size determined from more than 500 grains per alloy decreased from 51.6 to 18.1 μm, and the O-phase width decreased from 0.550 ± 0.191 to 0.194 ± 0.080 μm. RE_2_O_3_-type oxides formed, and their fraction increased with RE substitution. The EDS-derived apparent oxygen content decreased from 3.913 to 3.188 wt.% in grain-boundary α_2_ regions and from 1.630 to 1.065 wt.% in B2-matrix regions; these values support a redistribution trend but are not accurate bulk or phase-resolved oxygen measurements. At the same time, Nb decreased from 25 to 23 at.%; therefore, changes in α-Ti, α_2_, B2, and O phase fractions are attributed to coupled RE addition, oxygen partitioning, and Nb-content effects rather than to RE alone. The ImageJ particle-size results show a decrease in mean RE_2_O_3_ diameter from 10.19 ± 3.20 μm at 0.5RE to 5.04 ± 2.36 μm at 2.0RE, although particle number density and area/volume fraction remain unavailable.
3. At room temperature, the maximum *σ*_t,max_ was 572 MPa at 1.0RE and the maximum true fracture strain was 0.63% at 1.5RE. At 600 °C, the maximum *σ*_t,max_ was 634 MPa at 0.5RE, whereas the maximum true fracture strain was 0.98% at 1.5RE. Accordingly, 1.5 at.% RE is identified as providing the best overall balance in this five-alloy series because it combines near-maximum room-temperature strength with the highest true fracture strain at both temperatures; it is not the maximum-strength composition at 600 °C. The observed improvement is consistent with lower porosity, grain refinement, modified α_2_/O/B2 morphology, and RE-rich oxide formation. Oxygen redistribution and oxide-pinning effects remain proposed mechanisms that require future quantitative oxygen analysis and higher-resolution characterization for definitive confirmation.

## Figures and Tables

**Table 1:** Hyperparameter search ranges and selected configurations for the six regression models.

Model	Search Space (Main Parameters)	Selected Configuration
GB	n_estimators 100/200; learning_rate 0.01/0.1; max_depth 3/4/5; min_samples_split 2/5; min_samples_leaf 1/2	200; 0.1; 3; 5; 1
RF	n_estimators 100/200; max_depth 2–5; min_samples_split 2/5; min_samples_leaf 1/2; max_features 0.5/0.75/1.0; max_samples 0.8/1.0	200; 3; 2; 1; 0.75; 1.0
SVR	kernel linear/RBF; C 0.1–100; epsilon 0.01–0.2; gamma scale/0.001–1	linear; C = 1; epsilon = 0.01
EN	alpha 0.001–10; l1_ratio 0.1–1.0	alpha = 0.05; l1_ratio = 0.9
DNN	hidden layers (4, 4) to (16, 8, 4); activation tanh/ReLU; alpha 0.001–10	(4,4); tanh; alpha = 0.03
KNN	n_neighbors 1–10; weights uniform/distance; *p* 1/2	n_neighbors = 4; uniform; *p* = 1

**Table 2:** Crystallographic description of the principal phases discussed in the manuscript.

Phase	Representative Composition	Crystal Structure/Space Group	Identification in This Study
α-Ti	Ti-rich solid solution	hcp, P6_3_/mmc	XRD/Rietveld; peaks labeled Ti in Figure 6
B2	ordered Ti-(Al,Nb)	ordered bcc (CsCl type), Pm-3m	XRD/Rietveld; SEM morphology
α_2_	Ti_3_Al	hexagonal D0_19_, P6_3_/mmc	XRD/Rietveld; SEM contrast/morphology
O	Ti_2_AlNb	orthorhombic, Cmcm	XRD/Rietveld; acicular/lath morphology
RE_2_O_3_	La_2_O_3_/Ce_2_O_3_	A-type (trigonal), $P\bar{3}m1$	XRD + EDS-identified La-Ce-O-enriched regions

## Data Availability

The original contributions presented in this study are included in the article. Further inquiries can be directed to the corresponding author.

## Abbreviations

**Symbol/Abbreviation**	**Definition**
PM	powder metallurgy
CIP	cold isostatic pressing
ML	machine learning
DSC	differential scanning calorimetry
RE	rare earth (La-Ce addition in this study)
XRD	X-ray diffraction
SEM	scanning electron microscopy
EDS	energy-dispersive spectroscopy
GB	gradient boosting
RF	random forest
SVR	support vector regression
EN	elastic net
DNN	deep neural network
KNN	k-nearest neighbors
*σ*_t,max_	peak true stress
MSE	mean squared error
R^2^	coefficient of determination
A_1_, T_1_	DSC peak area and peak temperature in the 800–900 °C range
A_2_, T_2_	DSC peak area and peak temperature in the 1000–1100 °C range
α_2_	ordered Ti_3_Al phase
β	disordered bcc Ti-rich solid solution
B2	ordered bcc phase
O	orthorhombic Ti_2_AlNb phase
RE_2_O_3_	La/Ce-containing rare-earth-oxide product
EBSD	electron backscatter diffraction
TEM	transmission electron microscopy
SAED	selected-area electron diffraction
α-Ti	hexagonal close-packed Ti-rich solid solution
β-Ti	body-centered-cubic Ti-rich solid solution at elevated temperature
